# The Customized Heat Treatment for Enhancing the High-Temperature Durability of Laser-Directed Energy Deposition-Repaired Single-Crystal Superalloys

**DOI:** 10.3390/ma17225665

**Published:** 2024-11-20

**Authors:** Yimo Guo, Nannan Lu, Pengfei Yang, Jingjing Liang, Guangrui Zhang, Chuanyong Cui, Ting-An Zhang, Yizhou Zhou, Xiaofeng Sun, Jinguo Li

**Affiliations:** 1 School of Materials and Engineering, Northeastern University, Shenyang 110819, China; ymguo24h@imr.ac.cn (Y.G.); 2200687@stu.neu.edu.cn (P.Y.); 2 Shi-Changxu Innovation Center for Advanced Materials, Institute of Metal Research, Chinese Academy of Sciences, Shenyang 110016, China; jjliang@imr.ac.cn (J.L.); grzhang@imr.ac.cn (G.Z.); chycui@imr.ac.cn (C.C.); yzzhou@imr.ac.cn (Y.Z.); xfsun@imr.ac.cn (X.S.); 3 Key Laboratory for Ecological Utilization of Multimetallic Mineral, Ministry of Education, Northeastern University, Shenyang 110819, China; zta2000@163.net

**Keywords:** single-crystal superalloy, directed energy deposition, heat treatment, microstructure, high-temperature durability, microporous fracture

## Abstract

The high-temperature durability performance plays a crucial role in the applications of single-crystal (SX) superalloys repaired by laser-directed energy deposition (L-DED). A specialized heat treatment process for L-DED-repaired SX superalloys was developed in this study. The effect of the newly customized heat treatment on the microstructure and high-temperature mechanical properties of DD32 SX superalloy repaired by L-DED was investigated. Results indicate that the repaired area of the newly customized heat treatment specimen still maintained a SX structure, the average size of the γ′ phase was 236 nm, and the volume fraction was 69%. Obviously recrystallized grains were formed in the repair area of the standard heat treatment specimens, and carbide precipitated along the grain boundary. The size of the γ′ phase was about 535 nm. The high-temperature durable life of the newly custom heat treatment specimen was about 19.09 h at 1000 °C/280 MPa, the fracture mode was microporous aggregation fracture, and the fracture location was in the repair area. The durable life of the standard heat treatment specimen was about 8.70 h, the fracture mode was cleavage fracture, and the fracture location was in the matrix area. The crack source of both specimens was interdendrite carbide.

## 1. Introduction

Nickel-based superalloys are the primary materials for the manufacture of gas turbine and aero-engine turbine blades due to their excellent creep and fatigue corrosion resistance and good corrosion resistance [1]. To further optimize mechanical properties at high temperatures, such materials are usually cast into the single-crystal (SX) structure [2]. Unfortunately, surface defects like blade tip wear and crack initiation tend to form after the turbine blades have been working under severe service conditions. Considering the high replacement cost, it is of great economic and engineering significance to use suitable repair technology to repair damaged blades quickly and efficiently instead of replacing blades [3,4,5]. Traditional welding methods, such as tungsten inert gas (TIG) welding and gas-tungsten arc welding, tend to lead to the formation of stray grains (SGs) and cracks [6,7,8,9]. Therefore, advanced repair methods are essential to optimize the reuse of damaged SX parts.

The advent of laser-directed energy deposition (L-DED) provides a viable route for the repair of SX turbine blades, in which metal powder is injected into a melt pool created by a moving laser beam [10,11,12,13]. During the L-DED repair process, the heat input can be strictly controlled, and the heat-affected zone is small. The repaired area will form a dense metallurgical bond with the matrix, which is difficult to flake off. When specific solidification conditions are met, epitaxial growth of cells/dendrites along the original orientation in the substrate occurs, and no stray grains (SGs) are formed. Therefore, the repaired area can inherit the crystal orientation of the blade matrix and produce a dendrite structure consistent with the matrix orientation, which provides a feasible method for the repair of SX blades [14,15].

Researchers have successfully repaired SX superalloy parts without cracks by L-DED [16,17,18,19]. The structure of L-DED-repaired SX superalloy consists of an as-cast matrix area and a repaired area, accompanied by the fusion lines. In order to obtain excellent mechanical properties, the DED-repaired SX superalloy must be heat-treated before being returned to service [20,21]. However, most of the heat treatment studies of L-DED-repaired superalloys focus on the microstructure evolution and mechanical properties of the repaired area, and few studies have mentioned the matrix, which is also a part of L-DED-repaired SX superalloy. The deterioration of the matrix by heat treatment will also cause fracture during the mechanical properties test. Therefore, it is necessary to formulate a specific heat treatment process for different structures in the repair area and matrix area of the L-DED-repaired SX superalloy.

Unfortunately, till now, most researchers have usually used the standard heat treatment of as-cast SX superalloys to strengthen L-DED-repaired SX superalloys [22]. These standard heat treatment regimens have been developed for as-cast superalloys. It is evident that the standard heat treatment regimens for L-DED-repaired SX superalloys are not an optimal approach. The standard heat treatment of as-cast SX superalloy is generally composed of solution treatment and aging treatment [23]. L-DED is a process of rapid melting and solidification, which leads to a high level of residual tensile stress in the repaired area [24]. Residual stress will directly affect the geometric accuracy and mechanical properties of components. At present, it is reported that several research teams have detected residual stress in the components manufactured by L-DED [25,26]. For L-DED-repaired SX superalloy, excessive residual stress usually leads to recrystallization or even strain aging cracks in the repaired area during subsequent heat treatment. To avoid such problems, stress relief annealing is usually used as the first step of heat treatment to release residual stress, followed by other heat treatment steps.

Solution treatment can eliminate the eutectic phase, reduce element segregation, and release residual stress [27]. However, the high temperature of the solution treatment has a large impact on the structure of the matrix area. Local heat treatment of the repaired area may be the solution to this situation. Bian et al. [28,29,30] performed vacuum heat treatment and local heat treatment for laser melting deposition GH4169 at the same time and tested the high-temperature properties of the two heat treatment specimens. The results showed that the high-temperature tensile strength and yield strength of the local heat treatment specimens were slightly lower than those of the vacuum heat treatment specimens. In addition to the problem of damaging the high-temperature mechanical properties, the superalloy components are mostly aircraft combustion chamber and turbine end components in practical engineering applications, with uneven size and irregular shape, and it is difficult to achieve local heat treatment of these components. If solution treatment is carried out on the whole component, the high temperature of solution treatment will re-dissolve the γ′ phase in the as-cast matrix, and the γ′ phase precipitated again is far from the state before solution treatment, resulting in a significant decline in the mechanical properties of the matrix.

Therefore, it is necessary to reformulate the heat treatment regimen of DED-repaired SX superalloy. In this paper, a specific heat treatment process for L-DED-repaired SX superalloy was proposed. The new customized heat treatment process consists of an annealing treatment and an aging treatment. Optical microscopy (OM), scanning electron microscopy (SEM), and electron backscattering diffraction (EBSD) were used to observe the microstructure of the specimens after standard heat treatment and newly customized heat treatment. Then, two different heat treatment specimens were tested for high-temperature durability, and the failure reasons were analyzed. This work established a materials science foundation for further optimization of the process and regulation of post-heat treatment in the manufacture of L-DED-repaired SX superalloy parts.

## 2. Materials and Methods

### 2.1. Materials and Specimen Preparation

**Figure 1:**
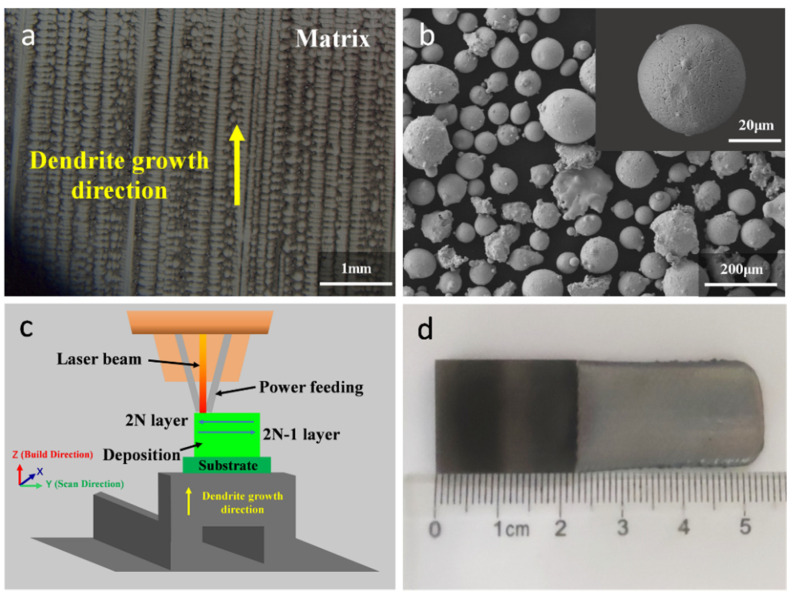
(**a**) Microstructure of cast DD32 SX superalloy substrate; (**b**) morphology of DD32 powder; (**c**) schematic diagram of additive manufacturing of SX superalloys by L-DED process; (**d**) thin-walled specimen repaired by L-DED.

The second-generation SX superalloy DD32 was used as the substrate and powder for the L-DED test. Its chemical composition (wt.%) is listed in Table 1. The DD32 SX superalloy substrate was prepared by high-rate solidification (HRS) directional solidification technology. The process parameters are as follows: pouring temperature is 1550 °C, the temperature in the upper zone of the holding furnace is 1520 °C, the temperature in the lower zone of the holding furnace is 1550 °C, and the pulling speed is 6 mm/min. The dendrite direction of the as-cast substrate is parallel to the crystal direction [001], and its microstructure is shown in Figure 1a. Before the repair experiment, to homogenize the element segregation between the dendrite core and the interdendritic region, the cast substrate was heat treated with 1290 °C × 4 h/AC + 1280 °C × 4 h/AC solution treatment [31]. All the substrates were sanded with 180 mesh sandpaper and subsequently cleaned with ethanol. The substrate was cut to 18 mm long, 22 mm high, and 2.5 mm wide by wire cutting. The powder was prepared by argon atomization and was spherical. The size of the DD32 powders was 50–150 μm, as shown in Figure 1b.

### 2.2. L-DED-Repaired Equipment and Process

The experimental L-DED system delivered powder by a coaxial nozzle, and the L-DED experiment was adopted with a CO_2_ laser with a maximum laser power of 5 kW and a spot size of 2 mm. All deposition experiments were performed in an argon atmosphere with oxygen content below 300 ppm. As shown in Figure 1c, the substrate is placed on a horizontal platform. In this work, the L-DED repairing experiments were performed at a laser power of 800 W, a scanning velocity of 5 mm/s, and a powder feeding rate of 4.0 g/min, as shown in Table 2. The size of the DD32 SX superalloy repaired by L-DED is 18 mm in length, 53 mm in height, and 2.5 mm in thickness, as shown in Figure 1d, and it consists of two parts: repaired area and matrix area.

**Figure 2:**
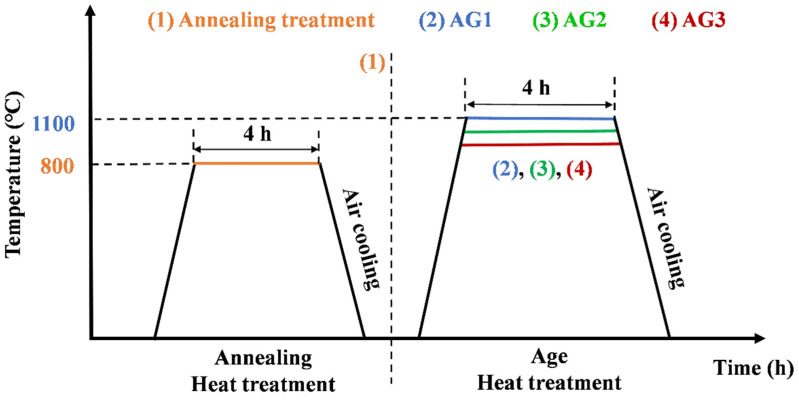
Schematic diagram of heat treatment process of the L-DED-repaired DD32 SX superalloy.

After the L-DED-repaired experiment, the as-deposited specimens were subjected to annealing treatment, followed by air cooling. In our previous work, it has been confirmed that 800 °C/4 h, AC is an optimal annealing treatment process for L-DED-repaired SX superalloy [32]. Therefore, this process is used as the first heat treatment step for L-DED-repaired SX superalloy. Subsequently, the specimens were subjected to aging treatment. The aging treatment process is shown in Table 3. The schematic diagram of heat treatment is shown in Figure 2.

### 2.3. Microstructural Observations and Mechanical Properties Testing

The microstructures of the as-deposited and the heat treatment specimens were characterized by using OM and SEM. All specimens for OM and SEM were mechanically polished and chemically etched with 20 g CuSO_4_ + 100 mL HCl + 80 mL H_2_O solution. The EBSD was used to analyze the crystal orientation of the as-deposited and heat treatment specimens. The EBSD specimens were prepared using a mechanical polishing method. The TSL OIM Analysis 7 software was used to analyze the ESBD data. The Image-Pro Plus 7 software was used to measure the size and volume fraction of γ′.

**Figure 3:**
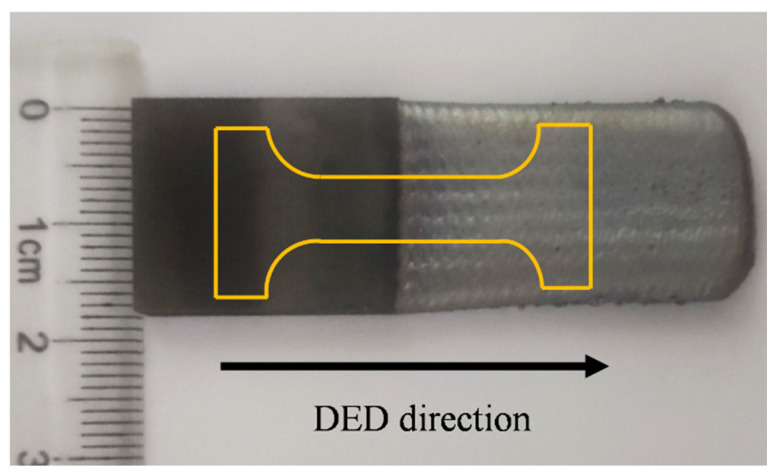
The specimens for high-temperature durability tests.

High-temperature durability is one of the important properties of SX superalloy. Therefore, the high-temperature durability of the L-DED-repaired SX superalloy was tested in this work. As shown in Figure 3, half of the area was repaired area and half of the area was as-cast SX superalloy. After heat treatment, the specimens were processed by wire cutting. The surface and sides of the specimen were polished by sandpaper. The high-temperature durability test was carried out at 1000 °C/280 MPa under the experimental conditions of a creep-testing machine until fracture.

## 3. Results

### 3.1. Observation of As-Deposited Microstructure

**Figure 4:**
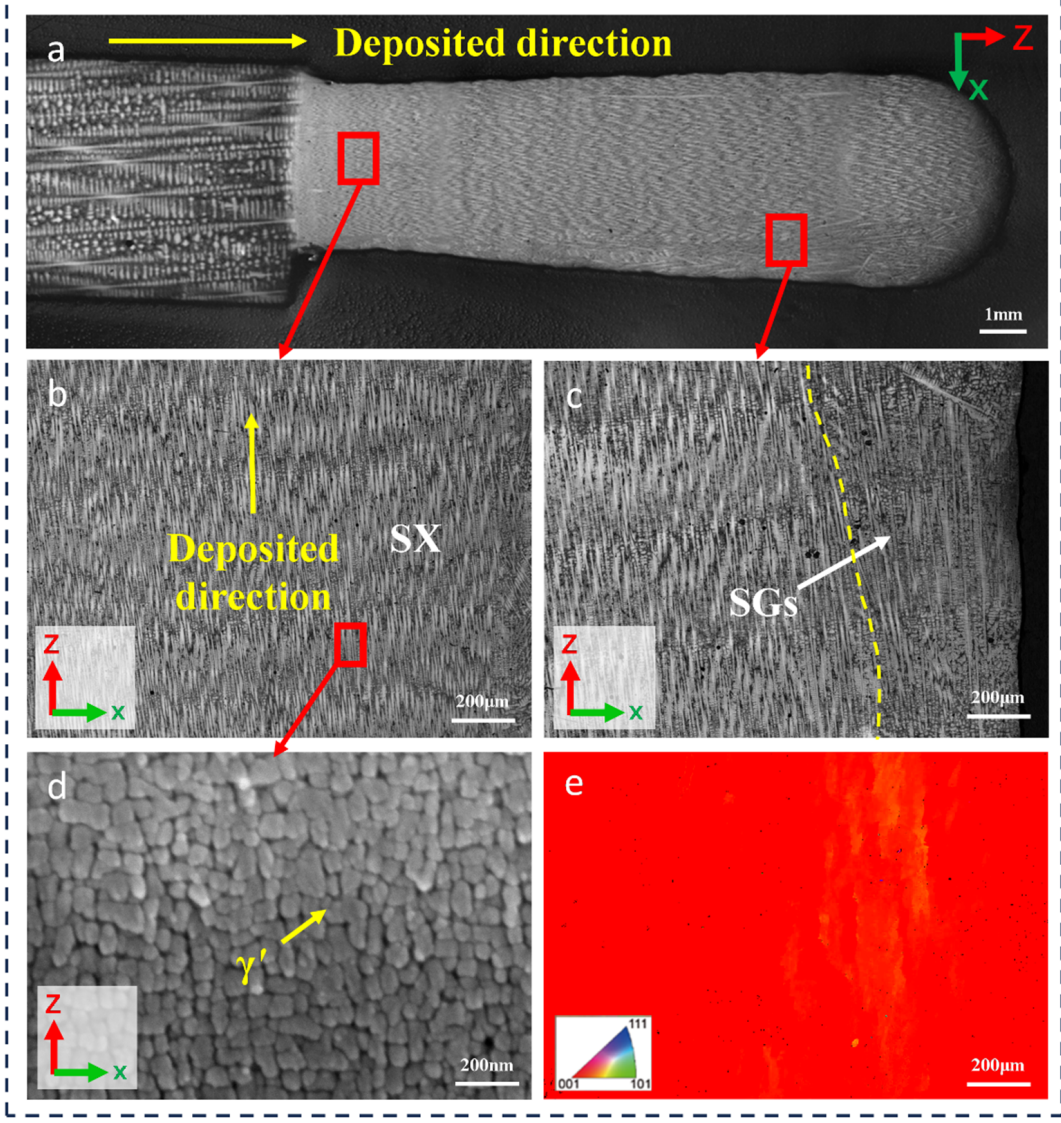
(**a**) The OM of DD32 SX superalloy repaired by L-DED; (**b**) the morphology of the bottom of repaired area; (**c**) the morphology of the top of repaired area; (**d**) the γ′ phase of repaired area; (**e**) EBSD mapping of the IPF image of (**b**).

The microstructure of the cross-section (perpendicular to the scanning direction) of the L-DED-repaired DD32 SX superalloy is shown in Figure 4a. No microcracks were found at the fusion interface and repaired area. As evident in Figure 4a,b, it can be observed that the microstructure of the repaired area is a continuous growth of microdendrites from bottom to top. Crystallographic analysis was performed on the repaired area of L-DED-repaired DD32 SX superalloy using EBSD characterization. As shown in Figure 4e, the repaired area exhibited a prominent epitaxial growth in the [001] direction, consistent with the SX substrate.

As the developed columnar crystals continued to grow upward, SGs gradually formed on both sides of the repaired area, as shown in Figure 4c. These SGs disrupted the growth orientation of the columnar dendrite, with the appearance of different dendrite growth directions. The formation of SGs is closely related to the solidification process [33]. With the increasing deposition height, the temperature gradient continues to decrease, and finally, the formation of SGs is promoted. This phenomenon can also be observed in other nickel-based superalloys and stainless steels manufactured using additive manufacturing techniques [34,35,36,37]. By higher-magnification SEM, as shown in Figure 4d, the γ′ phase of the repaired area of L-DED-repaired DD32 SX superalloy was approximately spherical, with a size of about 55 nm and a volume fraction of about 67%.

### 3.2. Design of Aging Treatment Process

After annealing treatment, the repaired area of L-DED-repaired DD32 SX superalloy still maintained the SX structure, and the γ′ phase of the repaired area was coarsened significantly [32]. However, the shape of the γ′ phases was still spherical, and the size of the γ′ phases was still small compared to the as-cast SX alloy. Therefore, aging treatment is necessary.

**Figure 5:**
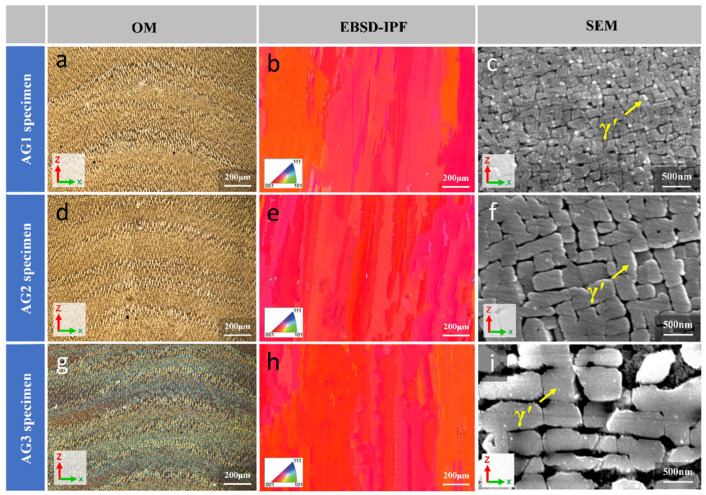
The OM of AG1, AG2, and AG3: (**a**,**d**,**g**); the EBSD mapping of the IPF image of AG1, AG2, and AG3: (**b**,**e**,**h**); The γ′ phase of AG1, AG2 and AG3: (**c**,**f**,**i**).

Figure 5a,d,g shows the OM image of the repaired area of AG1, AG2, and AG3 after heat treatment with different processes. It can be seen that after the aging treatment with different temperatures, all the alloys still maintained the SX structure, and no recrystallization formed. As shown in Figure 5b,e,h, the results of EBSD characterization also confirm the SX structure again. To further analyze the effect of the aging treatment on the microstructure of L-DED-repaired DD32 SX superalloy, AG1, AG2, and AG3 were observed by SEM. The results show that after the aging treatment, the microstructure of the repaired area was composed of γ and γ′ phases, with no secondary γ′ phase precipitation.

As shown in Figure 5c,f,i, γ′ phase size coarsened significantly with increasing aging temperature. The size and volume fraction of γ′ phase in the repaired area are the important parameters to analyze how the microstructure was influenced by the aging treatment temperature quantitatively. The γ′ phases of the AG1(900 °C) specimen were about 118 nm, the AG2(1000 °C) specimen was about 236 nm, and the AG3(1100 °C) specimen was about 456 nm, as shown in Figure 5c,f. With the aging temperature increasing to 1100 °C, the γ′ phase further coarsened, and the γ′ phase showed obvious rafting characteristics. The γ matrix channel increased significantly in terms of width, and some γ matrix channels disappeared, resulting in the merging of adjacent γ′ phases, as shown in Figure 5i. These rafting characteristics were considered to be unfavorable to the mechanical properties of superalloys [38].

With the aging temperature increasing from 900 °C to 1100 °C, the volume fraction of the γ′ phase was 71%, 69%, and 64%, respectively, as shown in Figure 5c,f,i. SX superalloy will have excellent high-temperature performance when the size of the γ′ phases is about 300 nm [23]. Therefore, after combining the influence factors such as the size, volume fraction, and morphology of the γ′ phase, AG2 was finally selected as the aging treatment process of the L-DED-repaired DD32 SX superalloy.

### 3.3. The Microstructure of the Standard and Newly Customized Heat Treatment Specimens

**Figure 6:**
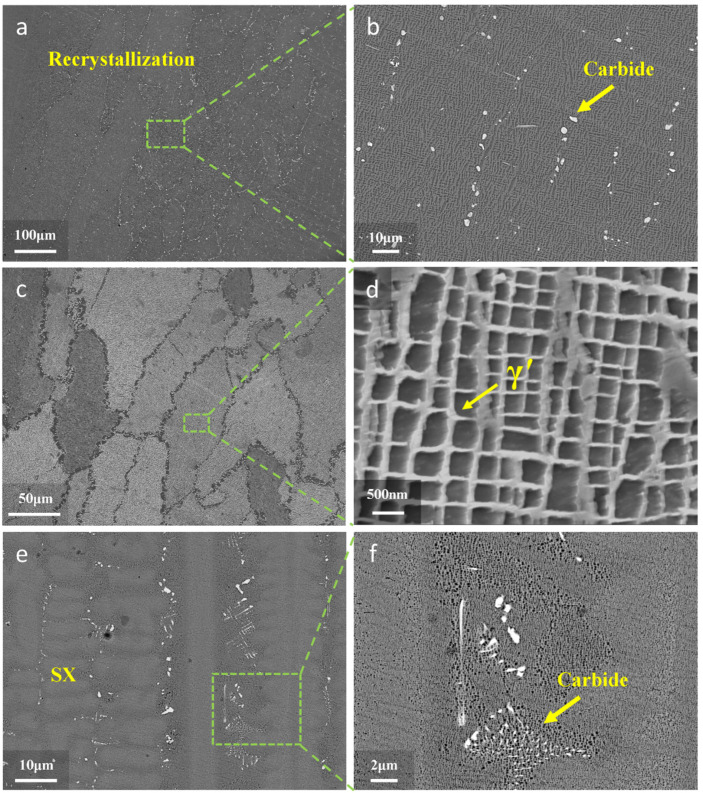
(**a**) The SEM image of repaired area of standard heat treatment specimen; (**b**) enlargement of (**a**); (**c**) recrystallized grains of repaired area of standard heat treatment specimen; (**d**) enlargement of (**c**); (**e**) the microstructure of repaired area of newly customized heat treatment specimen; (**f**) enlargement of (**e**).

For comparison with the newly customized heat treatment process (800 °C × 4 h/AC + 1000 °C × 4 h/AC), the standard heat treatment process of as-cast DD32 SX superalloy (1280 °C × 4 h/AC + 1290 °C × 4 h/AC + 1150 °C × 4 h/AC + 870 °C × 24 h/AC) was also performed for L-DED-repaired DD32 SX superalloy. The microstructure of repaired area of the standard heat treatment specimen is shown in Figure 6a,b. Because the specimens had not undergone annealing treatment, a large area of recrystallization appeared in the repaired area. It was further observed that the carbide morphology of the repaired area was granular and precipitated along the grain boundary after standard heat treatment.

Figure 6d shows the morphology of the γ′ phase of the repaired area of the standard heat treatment specimen. It can be observed that the size of the γ′ phase was about 535 nm, which was much higher than the newly customized heat treatment specimen (236 nm). The volume fraction of the γ′ phase decreased significantly, by about 60%, which was much lower than that of the newly customized heat treatment specimen.

After the newly customized heat treatment regimen, as shown in Figure 6e,f, the repaired area still contained a SX structure, and the carbides were distributed in the interdendrite region, with their morphology resembling fine fishbone [39].

### 3.4. High-Temperature Durability Test

Then, the newly customized heat treatment and standard heat treatment specimens were tested at 1000 °C/280 MPa for high-temperature durability. The results show that the average high-temperature endurance life of the newly customized heat treatment specimen was 19.09 h, and that of the standard heat treatment specimen was 8.70 h, which was about 55% lower than that of the newly customized heat treatment specimen, as shown in Table 4.

## 4. Discussion

Previous experimental results showed that the newly custom heat treatment significantly improved the high-temperature durability compared to the standard heat treatment. This section will discuss its basic mechanism.

### 4.1. Size and Morphology of γ′ Phase

**Figure 7:**
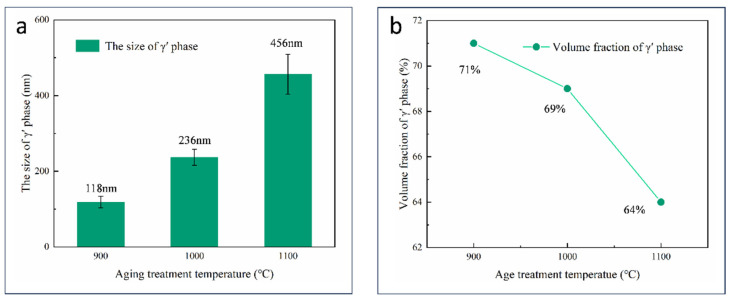
(**a**) The size of γ′ phase of AG1, AG2, and AG3; (**b**) the volume fraction of γ′ phase of AG1, AG2, and AG3.

The γ′ phase is the most important strengthening phase of nickel-based superalloys, and its volume fraction, size, morphology, and distribution have a decisive effect on the mechanical properties of nickel-based superalloys. The size and volume fraction of γ′ phases were measured for specimens, as depicted in Figure 7a,b. It can be seen that with the rise in aging temperature, the size of the γ′ phases gradually increased, while the volume fraction gradually decreased.

Mitchell et al. [40] proposed that the growth of the γ′ phases during the annealing process was mainly determined by the movement of solute atoms, in accordance with the Arrhenius relation [41]:

(1) $$D=D_{0}\exp\left(-\frac{Q}{RT}\right)$$

where *D* is the diffusion coefficient, $D_{0}$ is the diffusion constant, *Q* is activation energy, *R* is the ideal gas constant, and *T* is the temperature. $D_{0}$ and *Q* are temperature independent, depending on the composition and crystal structure of the alloy, and their values will not change with the aging temperature, so *D* is only related to *T*.

With an increase in the aging temperature, the diffusion coefficient also increased. Therefore, under the action of solute atom diffusion at high temperature, the γ′ phase is progressively coarsened. The shift in the primary morphology of the γ′ phases evolving from spherical to cuboidal was influenced by a combination of elastic strain energy and surface energy [41,42]. For the as-deposited specimen, the γ′ phases were spherical-shaped, with an approximate diameter of 55 nm, and the surface energy was the main factor affecting the morphology [42]. Nevertheless, as the age treatment process carried on and the γ′ phases coarsened, elastic strain energy increased and replaced the surface energy as the predominant factor affecting the morphology [43]. Therefore, with increasing age treatment temperature, the γ′ phases transitioned from a spherical to a cuboidal shape. The driving force of γ′ phase rafting is the reduction of interface energy and elastic strain energy, and γ′ phase rafting will inevitably reduce the interfacial energy of the alloy [44]. The directional coarsening direction of the γ′ phase is affected by the internal stress, due to no external stress during the aging treatment process [45].

### 4.2. Fracture Mechanism Analysis

**Figure 8:**
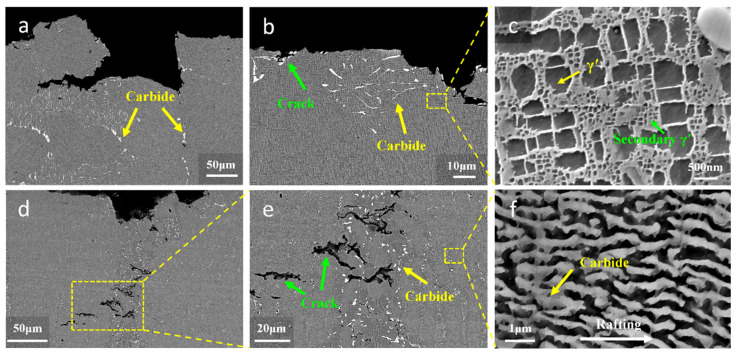
Longitudinal cross-section of standard heat treatment fracture specimen: (**a**) fracture location; (**b**) the morphology of cracks and carbides; (**c**) local amplification of (**b**). Longitudinal cross-section of newly customized heat treatment fracture specimen: (**d**) fracture location; (**e**) local amplification of cracks and carbides; (**f**) local amplification of (**e**).

The fracture surfaces and side surfaces of the high-temperature durability specimens were characterized using SEM to conduct a detailed investigation of the fracture mechanism. Figure 8 shows the longitudinal-section microstructures near the stress-ruptured surface of the high-temperature durability specimens. Through the analysis of dendrite morphology and carbide distribution, it can be observed that the fracture of the newly customized heat treatment specimens occurred in the repaired area under the 1000 °C/280 MPa high-temperature durability, as shown in Figure 8a–c. The fracture of the standard heat treatment specimens occurred in the matrix area, as shown in Figure 8d–f. The different fracture locations of the L-DED-repaired SX superalloy proved that the high-temperature durability property in the repaired area and the matrix area were different after different heat treatment processes.

As shown in Figure 8a,b,d,e, the longitudinal cracks of the fracture specimens were perpendicular to the applied stress direction, and the cracks of both specimens originated at the interface of carbides and the γ′ phase. As shown in Table 1, the DD32 SX superalloy contains 0.12~0.18% C element, and a large number of carbides were formed in the alloys prepared by directional solidification and additive manufacturing [46]. Ci et al. [47] confirmed that the carbides in DD32 were MC-type carbides. According to the composition of DD32 SX superalloy, common MC carbides included NbC, TaC, MoC, WC, etc. [48]. However, the carbide was hard and brittle and was weakly bound to the matrix [44]. The thermal expansion coefficient between the carbide and the γ phase matrix was significantly different. Therefore, the stress concentration easily led to the fracture of the carbide particles or the interface cracking between the carbide and the γ phase matrix. Although a large number of carbides were distributed in the microstructure of the two specimens, the morphology of the carbides was different. The carbides in the standard heat treatment specimens were short rod-shaped, while the carbides in the newly customized heat treatment specimens were granular.

By observing the longitudinal section of the fracture, it can be confirmed that the standard heat treatment specimens fractured in the heat-affected zone. Figure 8c shows the enlarged image of Figure 8b and shows that the volume fraction of the γ′ phase in the heat affected zone was relatively low, approximately 47%, and the cubic shape of the γ′ phase was poor. Meanwhile, a large number of secondary γ′ phases appeared here. As shown in Figure 8f, obvious raft characteristics were observed in the longitudinal fracture section microstructure of the newly customized heat treatment specimen. The direction of rafting is perpendicular to the direction of applied stress, and rafting originates from the diffusion of elements in the opposite direction of the stress gradient [49]. The stress gradient is determined by both the alloy mismatch and the applied stress conditions. Rafting occurs when the stress level is higher than the minimum stress level required for rafting [50].

**Figure 9:**
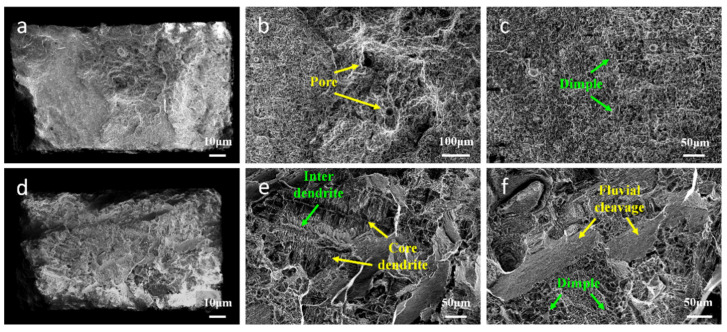
(**a**) The fracture surface of newly customized heat treatment fracture specimen; (**b**) the morphology of pore; (**c**) the morphology of dimple; (**d**) the fracture surface of standard heat treatment fracture specimen; (**e**) dendrite morphology; (**f**) cleavage fracture characteristics.

As evident in Figure 9, the fracture surfaces of the two specimens were observed by SEM. It can be seen that the fracture behavior of different specimens was significantly distinct one with another. The newly customized heat treatment specimen’s fracture surface presented numerous dimples and pores, and the fracture mode was typically micropore aggregation fracture, as shown in Figure 9a–c. Apparently, the solidified pores were ready-made micropores. A pore is a common metallurgical defect in additive manufacturing [51,52]. It is mainly produced by the involvement of unmelted powder or gas [53]. The unmelted powder is mainly related to the insufficient input of laser energy. When the energy density is low, the laser intensity of the penetrating powder is relatively reduced, resulting in local powder particles being unable to melt fully, thus forming elongated holes or irregular pores along the laser scanning direction [53]. Gas entrapment mainly results from the presence of argon gas within the hollow powder particles and the material vapor generated by the high laser energy, which becomes trapped in the molten pool. The formation of pores will directly affect the density of the alloy, reduce the hardness of the alloy, and have an adverse effect on the mechanical strength of the alloy during long-term service [54,55]. With the increase in temperature and imposed external stress, the micropores may aggregate and grow, thus forming the source of crack initiation [56].

The standard heat treatment specimens showed evident dendrite characteristics on the fracture surface, as well as the presence of dimples, as shown in Figure 9d–f. Subjected to standard heat treatment, the fracture mode of the specimens was cleavage fracture, approved by the fluvial form. The MC carbide in the matrix region was fragmented and distributed along the fracture surface. The interdendrite region became the crack source, due to MC carbides being mainly distributed in the interdendrite region.

### 4.3. Effect of Heat Treatment Process on High-Temperature Durable Life

Based on the previous analysis, it can be derived that the standard heat treatment specimens fractured in the matrix area and the newly customized heat treatment specimens fractured in the repaired area. The high-temperature durable life of the newly customized heat treatment specimens was significantly higher than that of the standard heat treatment specimens. These results indicate that the standard heat treatment will considerably reduce the high-temperature performance of cast matrix and eventually result in fracture.

**Figure 10:**
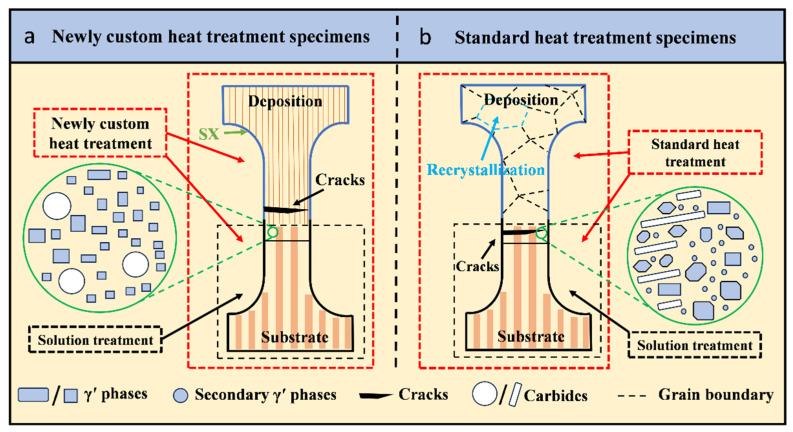
Schematic diagram of heat treatment and microstructure evolution: (**a**) newly customized heat treatment specimen, (**b**) standard heat treatment specimen.

Before the repair of SX superalloy, the as-cast SX matrix underwent solid solution treatment to achieve homogenization between the dendrite core and the interdendritic region. Meanwhile, as shown in Figure 10a, the DD32 SX superalloy adopted a double solution process (1280 °C/4 h, AC + 1290 °C/4 h, AC). After repairing, the SX superalloy was subjected to standard heat treatment (1280 °C/4 h, AC + 1290 °C/4 h, AC + 1150 °C/4 h, AC + 870 °C/24 h, AC). Multiple high-temperature solid solution treatments will cause repeated degrading of the γ′ phase in the heat-affected zone of the matrix area. It results in the morphology, size, and volume fraction of the γ′ phase not reaching the optimal state (Figure 8c), ultimately leading to the heat-affected zone at the matrix becoming the weak point of the specimens. Meanwhile, during multiple solid solution treatments, a secondary or even a tertiary γ′ phase will form in the heat-affected zone, which can seriously deteriorate the high-temperature mechanical properties [20]. However, as shown in Figure 10b, the newly customized heat treatment specimens will not suffer such damage to the matrix as they have not undergone multiple solid solution treatments. In addition, compared to the granular carbides formed in the newly customized heat treatment specimens, the bonding strength between the short, rod-shaped carbides formed in the standard heat treatment specimens and the matrix is weaker [22]. The morphology of the carbide is also one of the main reasons for the relatively short high-temperature endurance life of standard heat treatment specimens.

From another perspective, a large area of recrystallization was formed in the repaired area of the standard heat treatment specimens, and recrystallization will significantly deteriorate the high-temperature performance of the SX superalloy [57]. This showed that the standard heat treatment regimen of DD32 SX superalloy was not suitable for L-DED-repaired DD32 SX superalloy. However, the newly customized heat treatment specimens fractured in the repaired area during 1000 °C/280 MPa high-temperature durability testing, which implies that the newly customized heat treatment (800 °C/4 h, AC + 1000 °C/4 h, AC) avoided the deterioration of matrix properties, and no recrystallization occurred in the repaired area. Consequently, the newly customized heat treatment process had the effect of extending the high-temperature durable life.

## 5. Conclusions

The thin-walled structure of DD32 SX superalloy was repaired by L-DED. The L-DED-repaired SX superalloy was produced with a newly customized heat treatment, and microstructure observation and high-temperature durability tests were implemented. The main findings are summarized as follows:

(1) By observing the size, volume fraction, and morphology of the γ′ phase after different aging treatments, the optimal aging treatment process was determined to be 1000 °C/4 h, AC. At this time, the average size of the γ′ phase was 236 nm and the volume fraction was 69%.
(2) After standard heat treatment, the repaired area formed obvious recrystallized grains; carbide precipitated along the grain boundary. The size of the γ′ phase of the repaired area was about 535 nm; the volume fraction was about 60%.
(3) The high-temperature durable life of the newly customized heat treatment specimen was about 19.09 h, the fracture mode was microporous aggregation fracture, and the fracture location was in the repaired area. The high-temperature durable life of the standard heat treatment specimen was 8.70 h, the fracture mode was cleavage fracture, and the fracture location was in the matrix area. The crack source of both specimens was carbide.

In the future, the effects of process parameters on the epitaxial growth of SX superalloys should be further validated through simulations, and a process window for SX repair free of SGs and cracks should be established. Additionally, optimization of the heat treatment process is crucial for achieving effective engineering of DED-repaired SX blades.

## Figures and Tables

**Table 1:** Main chemical composition of DD32 SX superalloy (mass fraction, %).

Ni	Co	W	Ta	Cr	Al	Mo	C	Re	Nb
Bal.	8.00–10.00	7.70–9.50	3.50–4.50	4.30–5.60	5.60–6.30	0.80–1.40	0.12–0.18	3.50–4.50	1.40–1.80

**Table 2:** Experimental parameters of L-DED-repaired equipment.

Laser Power	Scanning Speed	Power Feeding Rate
800 W	5 mm/s	4 g/min

**Table 3:** Age treatment parameters of DD32 SX superalloy repaired by DED.

Specimen Number	Processes
AG1	900 °C/4 h
AG2	1000 °C/4 h
AG3	1100 °C/4 h

**Table 4:** The mean high-temperature durability life for the newly customized heat treatment specimen and standard heat treatment specimen under 1000 °C/280 MPa.

Specimen	High-Temperature Endurance Life/h
Newly custom heat treatment	19.09
Standard heat treatment	8.70

## Data Availability

The raw/processed data required to reproduce these findings cannot be shared at this time as the data also forms part of an ongoing study.
